# Analysis of neonatal cardiac function in infants with and without patent ductus arteriosus

**DOI:** 10.1186/1532-429X-15-S1-P289

**Published:** 2013-01-30

**Authors:** Kathryn Broadhouse, Anthony N Price, Giuliana Durighel, Anna Finnemore, David J Cox, AD Edwards, Joseph V Hajnal, Alan Groves

**Affiliations:** 1 Imaging Sciences Department, MRC Clinical Sciences Centre, Imperial College, Hammersmith Hospital, London, UK; 2 The Centre for the Developing Brain, Imaging Sciences & Biomedical Engineering Division, St Thomas' Hospital, King's College, London, UK

## Background

**Figure 1:**
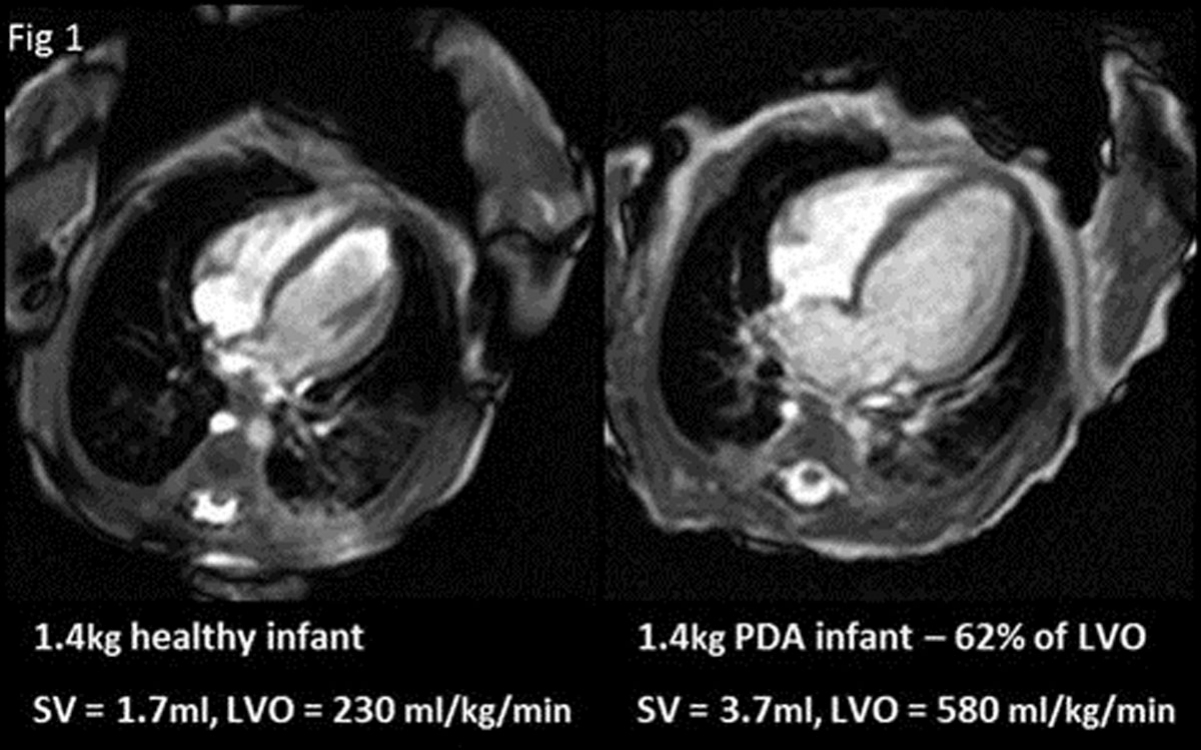
4 chamber view at end diastole in a 1.4kg control infant (left) and PDA (right) infant with a shunt volume of 62% of LVO.

Persistent PDA remains a common clinical presentation in preterm infants. We have already shown that high shunt volume increases LVO[1] and infants appear to have enlarged hearts (fig 1) but to what extent and their resultant function is yet unknown. The aim of this study was to quantify ventricular dimension and function in "healthy" neonates. Then compare PDA infants to this normative range to determine the impact of shunt volume.

## Methods

Scans were performed at 3T using pediatric and extremities coils. Infants were scanned with ear protection, routine monitoring and without sedation/anesthesia. Optimized 2D SSFP short axis 10-slice stacks[2] (resolution=0.5x0.5mm, slice=4mm) were acquired. Segmentation and quantification was carried out using freely available software Segment[3], the AHA model was used for wall analysis. Description of shunt volume quantification is described previously [1]. The ventricular dimension parameters LVmass, end diastolic volume (EDV), wall thickness and ventricular function parameters of stroke volume (SV), LVO, ejection fraction (EF), and fractional thickening (%thick) were quantified for both "healthy" (control) and PDA infants. The 2 groups were compared using unpaired student t-tests. Impact of shunt volume was analysed with multiple linear regression, p-values ≤ 0.05 being significant. Statistical analysis was carried out in Excel (Microsoft). Starlings curve was plotted to determine effect of PDA.

## Results

**Figure 2:**
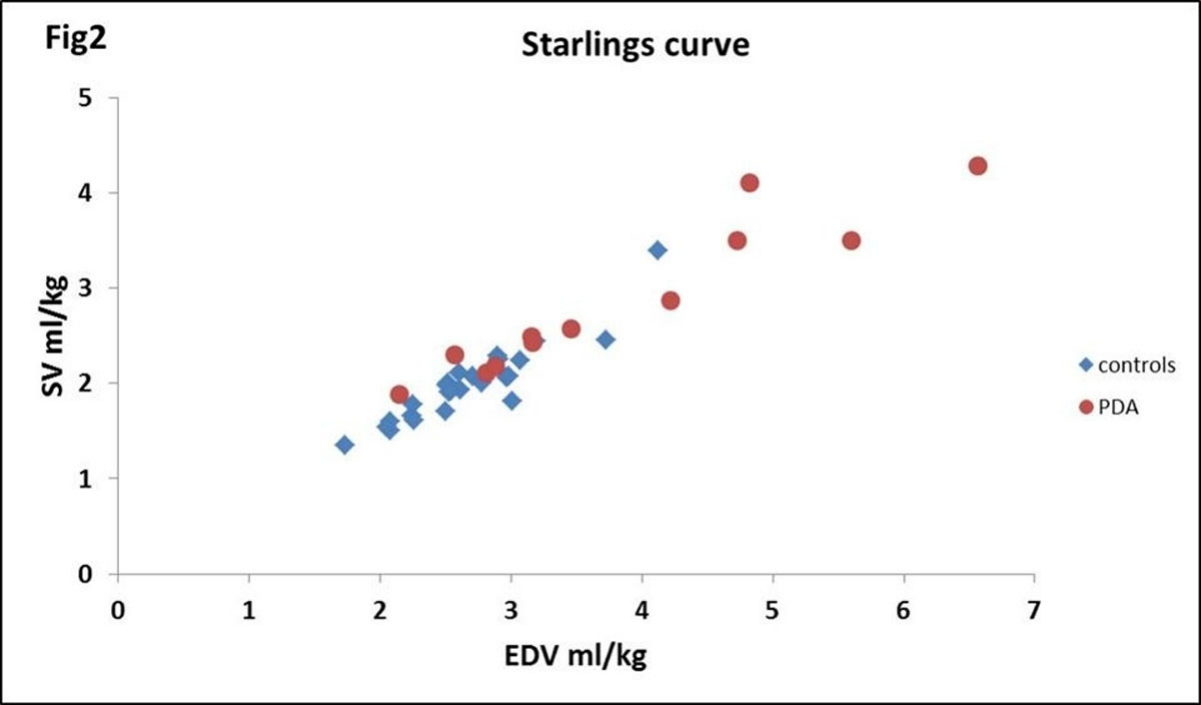
Population Starlings curve for controls and PDA infants

27 control infants median(range) corrected-GA 34+4(28+3-39+3)weeks, weight at scan 1730(790-3050)grams and 12 PDA infants (determined by echo prior to MRI) corrected-GA 29+5(27+3-36+1)weeks, weight at scan 1100(660-2400)grams were scanned. Shunt volume ranged from 7-74% of LVO[1]. T-tests showed significant difference in SV, EDV, ED wall thickness and LVmass between control and PDA infants when normalized by weight at scan. There was a significant association between shunt volume and increased LV mass when correcting for postnatal age and corrected-GA. However there was no significant difference in EF and %thick between the two groups (p-value=0.6 and 0.75). None of the PDA infants were over the Starlings curve (fig 2).

## Conclusions

Although LVO, EDV and LVmass are significantly increased in PDA infants, EF and %thick are not. In addition none of the PDA infants are over the Starlings curve. This would suggest that function is maintained in these enlarged hearts.

## Funding

MRC UK studentship
